# Commentary: Trehalose alleviates nephropathy in focal segmental glomerulosclerosis via the upregulation of the WT-1/EZH2 pathway

**DOI:** 10.3389/fphar.2026.1761856

**Published:** 2026-01-14

**Authors:** Fei Tian

**Affiliations:** Research Institute of Nephrology, Zhengzhou University, Zhengzhou, China

**Keywords:** fibrosis, focal segmental glomerular sclerosis, nephrotic syndrome, podocyte, renal disease

## Introduction

The recent study by Al-Asmakhi et al. provides compelling *in vivo* evidence for the therapeutic potential of trehalose in adriamycin-induced focal segmental glomerulosclerosis (FSGS) (Al-Asmakh et al., 2025). This work not only systematically demonstrates significant renoprotective effects but also innovatively shifts the mechanistic focus toward the regulation of the WT-1/EZH2 axis. As fellow researchers in nephrology, we applaud this conceptual advance. The purpose of this commentary is to engage constructively with these promising findings. We aim to discuss two key avenues for future work that could build upon this solid foundation: 1) strengthening the mechanistic evidence chain, and 2) contextualizing the findings within the broader, complex landscape of FSGS patient care to better gauge translational potential.

## An insufficient validation tier for β-catenin: a mechanistic gap needing addressal

**FIGURE 1:**
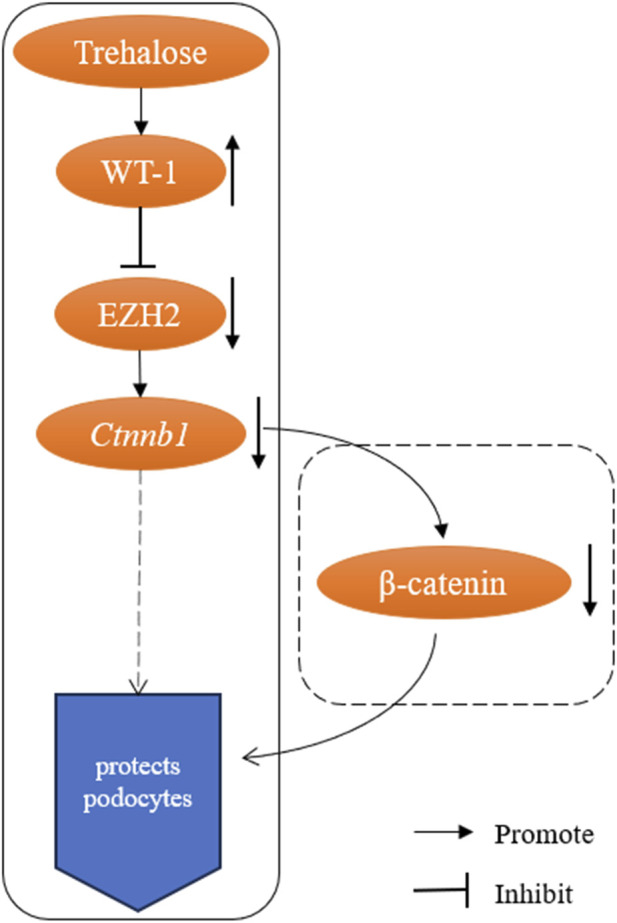
Schematic of the proposed trehalose-WT-1-EZH2-β-catenin pathway and the key validation gap. Solid boxes denote experimentally validated regulatory steps in the study; dashed boxes indicate hypothesized links requiring further validation, particularly the downregulation of β-catenin protein activity.

The core logical sequence of the study is: trehalose → upregulates WT-1 → inhibits EZH2 → downregulates β-catenin signaling → protects podocytes (Figure 1). The authors provide important preliminary support for this hypothesis by confirming the downregulation of β-catenin-encoding gene (*Ctnnb1*) mRNA via qPCR. However, in signal transduction research, mRNA changes are merely the beginning of the story, not its conclusion.

As the core effector of the canonical Wnt pathway, the pathogenic activity of β-catenin depends on protein stability, intracellular accumulation, and ultimately, nuclear translocation (Dai et al., 2009; Wan et al., 2017). Seminal work in the field, including that cited by the authors (Dai et al., 2009), unequivocally establishes that it is the activation of β-catenin at the protein level, not the mRNA level, that directly drives podocyte injury and proteinuria. Therefore, relying solely on transcriptomic data leaves us unable to confirm whether this critical pathogenic signal is effectively inhibited at the functional level. The absence of this evidence leaves the highly promising central mechanism of the “WT-1/EZH2/β-catenin” axis still in the realm of correlative inference, without fully establishing causality. Supplementing evidence at the protein level is a necessary and standard step to enhance the persuasiveness of this mechanistic model.

## Concrete validation pathways and their scientific value

To address the aforementioned gap and lend ultimate conviction to the proposed mechanism, we suggest the following direct and feasible experimental verification strategies: 1) Validation of Total Protein Level: Perform Western blot analysis on kidney tissue lysates to quantitatively detect the expression level of total β-catenin protein. This is the most direct method to verify whether transcriptional downregulation leads to reduced protein synthesis. 2) Validation of Functional Activity: Use immunofluorescence staining on kidney tissue sections to visually observe the subcellular localization of β-catenin (particularly its non-phosphorylated active form) within podocytes and glomeruli. Effective inhibition of the pathway by trehalose should manifest as a significant reduction in nuclear β-catenin signal. Alternatively, quantitative analysis via nuclear-cytoplasmic fractionation could be employed. These experiments do not negate the existing findings but rather adhere to the gold standard in the field for validating critical signaling pathways. Their completion would elevate the work from “discovering a promising correlation” to “elucidating a clear causal pathway,” thereby providing a more solid foundation for the field.

## Evaluating translational potential: benefits, patient subtypes, and unanswered clinical questions

Beyond mechanism, the exciting prospect of trehalose as a therapy invites a forward-looking discussion on its clinical viability. A balanced consideration of its potential benefits against inherent challenges is crucial for guiding future research.

A primary benefit is trehalose’s excellent safety profile as a natural compound, potentially offering a favorable side-effect paradigm compared to current immunosuppressive therapies. However, a key consideration is the model’s translation to human disease. The adriamycin-induced model robustly replicates podocyte injury, a final common pathway in FSGS. This suggests trehalose might hold particular promise for primary FSGS subtypes driven by podocytopathies. Its efficacy in secondary FSGS (e.g., due to obesity, hypertension), which involves more heterogeneous insults, remains an open and vital question for future investigation.

This leads to important limitations in the current study that affect clinical foresight. The acute injury model does not address long-term outcomes in chronic, progressive disease. Will trehalose prevent or reverse established sclerosis? Could it be used as a disease-modifying adjunct to existing therapies? Furthermore, its potential to prevent post-transplant recurrence, a major clinical hurdle, is unexplored. Addressing these questions in chronic models and combination therapy studies is a necessary next step to define trehalose’s realistic role in FSGS management.

## Discussion

The work by Al-Asmakhi et al. is a significant and stimulating contribution that successfully identifies a novel therapeutic candidate and a specific mechanistic pathway. Its importance lies in shifting the therapeutic strategy from supporting fundamental cellular processes like autophagy, towards actively regulating the key transcriptional program that determines podocyte fate via the WT-1/EZH2 axis. This provides a new logical framework for FSGS treatment: reinforcing podocyte identity through epigenetic modulation, rather than merely alleviating cellular stress.

From a methodological perspective, the study establishes a credible starting point for this novel hypothesis through a clear chain of *in vivo* efficacy and mechanistic evidence. The central challenge for its translational potential, however, stems from the inherent limitations of acute injury models in recapitulating the chronic progression and heterogeneity of human FSGS. Therefore, the critical next step in evolving trehalose from a ‘promising candidate’ to a ‘credible therapeutic option’ lies in validating its long-term efficacy and defining specific scenarios for its synergistic use with existing standard therapies, within more complex models such as chronic or secondary FSGS settings.

Our commentary seeks to extend the conversation it has ignited. By advocating for the integration of protein-functional validation, we hope to help fortify the mechanistic bedrock of this discovery. By thoughtfully considering patient applicability and long-term therapeutic scenarios, we aim to contribute to a more nuanced roadmap for its translational development. We are optimistic that pursuing these directions will clarify whether trehalose could become a standalone therapy, a safe combination agent, or a preventive strategy, ultimately enriching the arsenal against FSGS. We look forward to seeing the authors and the broader research community build upon this excellent work.
